# Supplementary material for: The association between new-use of antipsychotics and all-cause mortality in a cohort of patients with dementia in Argentina
Source: PLOS Ment Health. 2026 Feb 11;3(2):e0000554. doi: 10.1371/journal.pmen.0000554 (PMC12893566; doi:10.1371/journal.pmen.0000554)
Supplement: S1 Text — This file includes detailed definitions of dementia-related diagnostic terminology, psychotropic drug exposure, and technical analytical detail. (DOCX) [file pmen.0000554.s001.docx]

**S1 Text. Definition of study variables and R packages**

**Diagnosis of dementia as documented in the neurocognitive assessment report**

Diagnosis of dementia or equivalent terms: "neurodegenerative", "Major neurocognitive disorder", "Major cognitive disorder", "severe neurocognitive disorder", " Major cognitive disorder, moderate stage", “Alzheimer's disease”, "Parkinson's disease", “Lewy", " Neurocognitive disorder with Lewy bodies", "Frontotemporal dementia", "Pick's disease”, "Vascular dementia", "Vascular pattern", "Severe neurocognitive disorder probably associated with vascular disease" and “Mixed pattern”.

### **Psychotropic drugs exposure:**

We defined psychotropic use as the fulfillment of at least one psychotropic prescription at the start of the follow-up period

**Antidepressants:**

Selective serotonin reuptake inhibitors**:** Fluoxetine, Fluvoxamine, Escitalopram, Citalopram, Sertraline and Paroxetine

Tricyclic antidepressants (TCA): Amitriptyline, nortriptyline, clomipramine, Imipramine

Serotonin and noradrenaline reuptake inhibitors (SNRIs): Venlafaxine, duloxetine.

Other Dual-action antidepressants (DAA): Bupropion, mianserine, mirtazapine.

Others: trazodone, agomelatine.

**Benzodiazepines:** alprazolam, clonazepam, diazepam, bromazepam, lorazepam, midazolam, Oxazepam, Clorazepate and clobazam.

**Z-drugs:** zolpidem, zopiclone, eszopiclone.

**Antiepileptics:** phenobarbital, carbamazepine, valproic Acid, phenytoin, topiramate, levetiracetam, oxcarbazepine, zonisamide, lamotrigine, lacosamide, gabapentin and Pregabalin.

**Opioids:** Morphine, methadone, tramadol, codeine, oxycodone, fentanyl, nalbuphine and buprenorphine.

**R packages**

Analyses were conducted in R using the following packages and versions: tidyverse (2.0.0), lubridate (1.9.3), reshape2 (1.4.4), survival (3.7-0), rms (6.8-2), mice (3.19.0), survminer (0.4.9), purrr (1.0.2), and splines (4.4.1).
