# Supplementary material for: The association between new-use of antipsychotics and all-cause mortality in a cohort of patients with dementia in Argentina
Source: PLOS Ment Health. 2026 Feb 11;3(2):e0000554. doi: 10.1371/journal.pmen.0000554 (PMC12893566; doi:10.1371/journal.pmen.0000554)
Supplement: S1 Table — This table provides the cut-off scores used to identify cognitive impairment using the Mini Mental State Examination (MMSE). Thresholds are adjusted for both age and years of education. These values were applied when an explicit diagnosis of Major Neurocognitive Disorder was not documented in the clinical records. (DOCX) [file pmen.0000554.s003.docx]

**S1 Table. Mini mental State Examination score thresholds adjusted by age and education level.**

This table provides the cut-off scores used to identify cognitive impairment using the Mini Mental State Examination. Thresholds are adjusted for both age and years of education. These values were applied when an explicit diagnosis of Major Neurocognitive Disorder was not documented in the clinical records.

| **Education (years)** | **Age (years)** | | | | |
| --- | --- | --- | --- | --- | --- |
|  | <45 | 56-65 | 46-55 | 56-75 | >75 |
| < 5 | - | - | 24 | 24 | 22 |
| 5-7 | 27 | 27 | 26 | 26 | 25 |
| 8-12 | 27 | 27 | 27 | 27 | 25 |
| >12 | 27 | 27 | 27 | 27 | 26 |

Adaptation of Butman, J., Arizaga, R. L., Harris, P., Drake, M., Baumann, D., De Pascale, A., ... & Ollari, J. A. (2001). El “mini-mental state examination” en español. Normas para Buenos Aires. Rev Neurol Arg, 26(1), 11-15
