# Supplementary material for: The association between new-use of antipsychotics and all-cause mortality in a cohort of patients with dementia in Argentina
Source: PLOS Ment Health. 2026 Feb 11;3(2):e0000554. doi: 10.1371/journal.pmen.0000554 (PMC12893566; doi:10.1371/journal.pmen.0000554)
Supplement: S2 Table — This table lists standardized daily doses (SDD) for antipsychotic medications in older adults. (DOCX) [file pmen.0000554.s004.docx]

**S2 Table. Antipsychotics drugs included and minimum geriatric doses of antipsychotics**.

| **Antipsychotic** | **Standardized daily doses, in milligrams** |
| --- | --- |
| Atypical antipsychotics |  |
| Aripiprazole | 10 |
| Clozapine | 75 |
| Olanzapine | 5 |
| Quetiapine | 12.5 |
| Paliperidone | 2.5 |
| Risperidone | 1 |
| Ziprasidone | 40 |
| Typical antipsychotics |  |
| Chlorpromazine | 150 |
| Clotiapine | 100 |
| Haloperidol | 1 |
| Levomepromazine | 150 |
| Trifluoperazine¹ | 20 |

This table lists standardized defined doses (SDD) for antipsychotic medications in older adults.

1. When minimum geriatric doses were undefined (e.g., for trifluoperazine), WHO’s Defined Daily Doses were used as reference (see <https://atcddd.fhi.no/atc_ddd_index>).
