# Supplementary material for: The association between new-use of antipsychotics and all-cause mortality in a cohort of patients with dementia in Argentina
Source: PLOS Ment Health. 2026 Feb 11;3(2):e0000554. doi: 10.1371/journal.pmen.0000554 (PMC12893566; doi:10.1371/journal.pmen.0000554)
Supplement: S3 Table — This table depicts the proportion of participants with time-varying covariates since dementia diagnosis at different time points during follow-up. (DOCX) [file pmen.0000554.s005.docx]

**S3 Table.** Proportion of participants with time-varying covariates since dementia diagnosis.

| **Time, in months** | **Opioids** | **Benzodiazepines** | **Antidepressants** | **Antiepileptics** | **Z-drugs** | **Coronary disease** | **Cardiac failure** | **Insomnia** | **Anxiety** |
| --- | --- | --- | --- | --- | --- | --- | --- | --- | --- |
| 6 | 2.2 | 15.7 | 20.0 | 6.1 | 2.3 | 11.2 | 5.4 | 13.0 | 24.3 |
| 12 | 2.4 | 15.3 | 20.8 | 6.9 | 2.3 | 11.0 | 5.7 | 13.3 | 24.6 |
| 24 | 2.4 | 16.2 | 21.9 | 5.7 | 2.3 | 10.7 | 5.7 | 13.3 | 25.8 |
| 36 | 1.9 | 16.4 | 20.2 | 7.6 | 3.9 | 10.7 | 6.3 | 14.2 | 25.4 |
| 54 | 0.6 | 14.1 | 21.8 | 5.8 | 4.5 | 5.1 | 5.8 | 18.6 | 21.2 |

All numbers represent percentage points unless otherwise specified.
