# Supplementary material for: The association between new-use of antipsychotics and all-cause mortality in a cohort of patients with dementia in Argentina
Source: PLOS Ment Health. 2026 Feb 11;3(2):e0000554. doi: 10.1371/journal.pmen.0000554 (PMC12893566; doi:10.1371/journal.pmen.0000554)
Supplement: Attachment — Submitted filename: response_to_reviewers.docx [file pmen.0000554.s006.docx]

**Response to reviewers’ comments**

We appreciate the careful and thoughtful feedback provided by the reviewers of our manuscript. We believe that this feedback has greatly improved our paper. The reviewers’ comments appear in italic and have been assigned a label (C1, C2, C3…). Our responses to each comment have been assigned a corresponding label (R1, R2, R3…). Also, we have included a label if a change in the manuscript was performed (“Changes in Manuscript”).

**Reviewer #1**

**C1) This manuscript makes a valuable contribution to the limited literature on geriatric mental health in low- and middle-income countries by examining the association between antipsychotic initiation and all-cause mortality in a dementia cohort in Argentina. It is a relevant and timely topic. The work is generally methodologically sound, and the statistical approach seems appropriate, although reproducibility could be enhanced by providing access to the dataset and R code.**

**R1)** We thank the reviewer for their positive feedback and thoughtful comments. To promote transparency and reproducibility, we have published an R code on GitHub for readers who are interested in learning the technical details of analysis implementation. The code is published alongside data generated for the purpose of reproducing the methods of the main analysis, although it does not reflect real patients’ data.

**C2) DATA SOURCE**

**LINE 137: The authors did well to describe SDD, but it is not immediately clear how it was calculated in this study. If the number of days of supply of antipsychotics was missing from the pharmacy data, did the authors estimate from dosage instructions, or infer from refill intervals, etc.**

**R2)** We appreciate the reviewer’s comment. In this study, prescription duration was not available, so we focused our measurements on cumulative dose use. Because SDD represent the average or recommended daily dose of a medication, different amounts of SDD can be interpreted as a given number of days of supply if a standard dose is assumed each day. This may mislead readers into thinking we measured duration of supply rather than cumulative dose. To clarify this issue, we have now included a worked example illustrating how SDD were calculated and how they should be interpreted.

**Changes in manuscript:**

**Line 170-171:** remove: *“In other words, we calculated the total amount of SDD for all antipsychotics in filled prescriptions for study participants.”*

**Line 183,** remove: *“In this study, one SDD is defined as the recommended daily dose of an antipsychotic medication for the treatment of older adults. For example, in clinical practice, a patient would achieve 90 SDD of quetiapine exposure by taking 12.5 mg of quetiapine daily for 90 days.”*

**Line 171,** add: *“(…) the average or recommended daily amount of medication per day, (…)”*

**Line 178,** add: *“Although SDD do not measure duration of exposure, they can provide clinically meaningful interpretation to cumulative antipsychotic exposure. For example, a filled prescription for 30 tablets of quetiapine 25 mg corresponds to a cumulative dose of 750 mg (30 times 25). This quantity is then divided by the recommended dose for quetiapine in older adults (12.5 mg) (1), yielding 60 SDD. Hence, the filled prescription for quetiapine equals a cumulative dose of 60 SDD, which can be interpreted as the equivalent of 60 days of supply, assuming the recommended dose was taken each day.”*

**C3) STUDY POPULATION**

**LINE 140: Epidemiological studies on dementia in Latin America seem to favor the age of 65 years as a cutoff (Table 3, PMID: 29305437). It would be beneficial to clarify the rationale behind the authors' choice of 60 years in this study.**

**R3)** We thank the reviewer for this observation. The rationale for including individuals aged ≥60 years followed the clinical practice literature published by the Argentinian Society of Gerontology and Geriatrics (original name in Spanish: “Sociedad Argentina de Gerontología y Geriatría”)^1^ and is also in accordance with the World Health Organization’s initiative to promote healthy aging.^2^

1.	SAGG | Sociedad Argentina de Gerontologia y Geriatria. Accessed March 24, 2024. https://sagg.ar/

2.	Ageing and health. Accessed February 26, 2024. https://www.who.int/news-room/fact-sheets/detail/ageing-and-health

**C4) STUDY POPULATION**

**LINE 143: Since the authors used MMSE as a proxy for dementia in certain cases, it would strengthen the manuscript to include information about validation studies of this screening tool on the Latin American population at a minimum, or in Argentina if available.**

**R4)** We thank the reviewer for this suggestion. We have added references to validation studies conducted in Latin American populations, including Argentina, in the Methods section. These studies support the validity and cultural relevance of the MMSE as a cognitive screening tool among older adults in this regional context.

**Changes in Manuscript**

**Line 149,** add: “*The Spanish-language version of the MMSE has been validated in a previous study in Argentina, using a representative sample of 634 participants aged 30 to 94 years, stratified sex and educational level (Butman 2001). Similar normative data have also been reported in other Latin American populations, including Chile, supporting the regional applicability of this screening tool (Molina-Donoso 2023).”*

*New references:*

*1)Butman J, Arizaga RL, Harris P, Drake M, Baumann D, Morales MC, et al. El “Mini-Mental State Examination” en español. Normas para Buenos Aires. Rev Neurol. 2001;33(8):695-698.*

*2) Molina-Donoso M, González-Hernández J, Delgado C, Cancino V, Bello-Lepe S, Alonso-Sánchez MF, et al. Nuevos datos normativos para el Mini Mental State Examination (MMSE) en la población de personas mayores en Chile. Rev Med Chile. 2023 Nov;151(11):1464-1470. DOI: 10.4067/S0034-98872023001101464.*

**C5) OUTCOME**

**LINE 152: How different is “five years of follow-up” from “the end of the study period”? If there is a difference, when did the study period end?**

**OUTCOME**

**LINE 153: What does "disenrollment" from the HMO mean? Does it imply a loss to follow-up?**

**R5)** We thank the reviewer for commenting on this issue. We have added a brief clarification for each term to enhance readability.

**Changes in manuscript**

**Line 161,** add: *“(i.e., December 31, 2021)”*

**Line 162,** add: *“(i.e., loss to follow-up)”*

**C6)**

**SECONDARY AND SENSITIVITY ANALYSES**

**LINE 235: In the interest of reproducibility, can the R codes and packages used in the analysis be included in the supplementary table?**

**R6)** We thank the reviewer for the comment. We have included information on R packages that were used as suggested. Information on packages’ versions are also published in the public R code on GitHub.

**Changes in manuscript**

**Supporting information**, add:

S1 Text. Definition of study variables *and R packages used*

*Analyses were conducted in R using the following packages and versions: tidyverse (2.0.0), lubridate (1.9.3), reshape2 (1.4.4), survival (3.7-0), rms (6.8-2), mice (3.19.0), survminer (0.4.9), purrr (1.0.2), and splines (4.4.1).*

**C7) - A brief comment on how censoring was handled would improve clarity.**

**R7)** A one sentence clarification has been added to the methods section.

**Changes in manuscript**

**Line 213,** add: “C*ensoring was assumed to be independent (i.e., the mechanism of censoring is non-informative).”*

**C8) DISCUSSION**

**LINE 351: I suggest rephrasing the latter portion of this sentence because, although MMSE is widely used to stage dementia severity, it is technically a screening tool for cognitive impairment.**

**R8)** We appreciate the reviewer’s helpful comment. We have revised the sentence to better reflect the role of the MMSE.

**Changes in manuscript**

**Abstract section,** line 51 remove: “*dementia severity”.*

**Line 372,** add: “*However, we leveraged standardized assessments to identify dementia cases and mental health symptoms, including MMSE scores and depressive symptoms scales.”*

**C9) DISCUSSION**

**- A brief statement on the generalizability of these findings would be helpful.**

**R9)** We thank the reviewer for this suggestion. We have incorporated a brief comment on the generalizability of our findings to populations with dissimilar baseline risks and distribution of effect modifiers.

**Changes in manuscript**

**Line 389**, add: “*Finally, participants of this study were affiliated with a health maintenance organization and underwent cognitive testing for dementia. By requiring a cognitive test for inclusion, the study may have disproportionately enrolled individuals with more prominent symptoms, better healthcare access, or distinct health‑seeking patterns, either alone or in combination (Rojas-Saunero 2024). ). This may select patients who are not representative of the broader population of individuals living with dementia (45). In particular, our study population consisted largely of urban, middle‑class adults, and the findings may not be generalizable to rural populations or to groups with different socioeconomic characteristics or healthcare seeking behaviors.”*

**C10) In summary, this is a well-conceived and relevant study. Addressing the points above would further strengthen the manuscript's impact.**

**R10)** We thank the reviewer for the positive feedback.

**Reviewer #2**

**C1) This manuscript addresses antipsychotic safety in dementia patients. It examines mortality risk in older adults newly diagnosed with dementia in Argentina and finds an increased risk of death among antipsychotic users. With some revisions, this work would make a novel and important contribution to the literature.**

**R1)** We thank the reviewer for the positive feedback.

**C2) Consider specifying "new-use" in the title.**

**R2)** We have incorporated “new-use” in the title and through the manuscript.

**Changes in manuscript**

Change in title and text body.

**C3) In the abstract, confirm if it is okay to specify the exact location and name of the hospital that patients came from. “outpatients aged 60 years and older at the Hospital Italiano de Buenos Aires’ health maintenance organization in Argentina” could potentially constitute identifying information**

**R3)** We have removed the name of the hospital from the Abstract to avoid including potentially identifying information.

**Changes in the manuscript**

**Line 40,** re-write: “We conducted a retrospective cohort study among outpatients aged 60 years and older receiving care within a large health maintenance organization in Argentina”

**C4) line 83: it may be good to provide a range instead of an exact figure, as I’ve seen higher estimates: “dementia prevalence is 7% among adults aged ≥75 years"**

**R4)** We thank the reviewer for this suggestion.

**Changes in manuscript**

**Line 87,** add: “*In Latin America, dementia prevalence is 8% to 17.1% among adults (1), exceeding estimates from high-income countries*.”

**C5) in the introduction, consider discussing specific mechanisms linking antipsychotics to early mortality, if possible**

**R5)** We thank the reviewer for this suggestion. We have added a sentence in the introduction related to the specific mechanisms linking antipsychotics to early mortality, a topic we also revisited in the discussion.

**Changes in manuscript**

**Line 78,** add: *“Studies suggest that, in patients with dementia, antipsychotic use may exacerbate neurovascular and neurodegenerative processes, increasing susceptibility to hypoperfusion-related injury and contributing to disease progression through interactions with tau pathology, cerebral amyloid angiopathy and small vessel disease (Murray 2014). Clinical evidence from (…)”*

**C6) in the methods, consider discussing potential selection bias from requiring individuals who have “underwent an ambulatory neurocognitive assessment” (Line 141), even if it may be a prerequisite for dementia diagnosis**

**R6)** We thank the reviewer for highlighting this issue. We have elaborated on this potential source of bias by including the potential for selection or generalizability bias by restricting the population to those patients with dementia who underwent cognitive tests.

**Changes in manuscript**

**Line 389**, add: “*Finally, participants of this study were affiliated with a health maintenance organization and underwent cognitive testing for dementia. By requiring a cognitive test for inclusion, the study may have disproportionately enrolled individuals with more prominent symptoms, better healthcare access, or distinct health‑seeking patterns, either alone or in combination (Rojas-Saunero 2024). This may select dissimilar patients, and findings may not be generalized to populations with other baseline risks or distributions of effect modifiers (Rojas-Saunero 2024).”*

**C7) Line 167: in the methods, the choice of minimum geriatric doses may need better justification and include what this dosage is or the range it may constitute. It may also be good to specify what this “clinical practice” is and what standards are used to determine this minimum dosage**

**R7)** We thank the reviewer for commenting on this potential issue. We have now included references for previous research studies that also implemented SDD as calculated using this method.

**Changes in manuscript**

**Line 181**, add: “*(…)Minimum geriatric doses reflect routine geriatric prescribing practice, whereby older adults are treated with lower doses than younger populations to achieve clinical response while potentially reducing adverse events, as outlined in a renowned pharmacy reference, as outlined in a renowned pharmacy reference”*

**Line 180,** add: *“and they have been used in several studies previously (Gray 2015, Gray 2018, Hart 2019, Li 2024).”*

**C8) Line 188: For statistical analysis, I find there to be fairly sparse imputation details with just one sentence saying: ‘We handled missing data using multiple imputation by chained equations.”. Which variables had missing data and why were specific predictors chosen?**

**R8)** We thank the reviewer for the feedback. We have moved information about missing data from the results section to the methods section, and now we provide more details on the procedures to impute data.

**Changes in manuscript**

**Move from results to methods,** line 205: **“***Missing data were present in education (1.0%), Beck Depression Inventory II scores (15.0%), and MMSE scores (1.1%).”*

**Line 207,** add: “*using a maximum of 50 iterations*”

**Line 208,** add: “*The imputation model included variables that were also used in the analysis model (see below), and variables were selected iteratively until convergence was achieved.”*

**C9) in the results, the manuscript says, “In addition, we observed a higher risk of all-cause mortality among patients treated with typical antipsychotics (aHR = 4.41, 95% CI: 2.06 to 9.44) compared with non-users, and this risk was greater than that associated with atypical antipsychotics (aHR = 2.54, 95% CI: 2.80 to 3.58), compared with non-users”. Firstly, it would be good to note the small sample size and consider whether or not to include this statement given the weak power, as the typical antipsychotic group has only 27 person years and seem to potentially be as low as 13 patients? Furthermore, there is a discrepancy between table 2 and what is written in the paragraph. It appears the aHR for atypical antipsychotics should be 2.54 (1.80-3.58), not (2.80-3.58) that is written. Please double check all numbers in the manuscript to ensure alignment with the tables**

**R9)** We thank the reviewer for this comment. We agree with the reviewer; this specific analysis may deviate readers’ attention from the main objective of the study, and given the limitations in sample size, we have removed this analysis from the report. We also included a brief comment in the discussion section about this limitation.

**Changes in manuscript**

**Line 238,** remove: “*Finally, we compared the hazard of all-cause mortality across three groups: patients initiating typical antipsychotics versus non-users, patients initiating atypical antipsychotics versus non-users, and typical versus atypical antipsychotic users, adjusting for the same covariates as in the main analysis.”*

**Line 303, remove***: “In addition, we observed a higher risk of all-cause mortality among patients treated with typical antipsychotics (aHR = 4.41, 95% CI: 2.06 to 9.44) compared with non-users, and this risk was greater than that associated with atypical antipsychotics (aHR = 2.54, 95% CI: 2.80 to 3.58), compared with non-users. Compared with new-use of atypical antipsychotics, new-use of typical antipsychotics was associated with 1.74 times (95%CI: 0.80 to 3.76) higher hazard of all-cause mortality. Finally, our results remained consistent in sensitivity analyses (Table 2).”*

**Table 2,** remove**:** analysis results from typical and atypical antipsychotics.

**Line 388,** add: *“Fourth, due to limitations related to sample size (…) or medication classes (i.e., “typical” or “atypical”)”*

**C10) at times there seems to be abbreviation inconsistency with regards to “aHR” vs “adjusted hazard ratio”. Please ensure consistency throughout except the first instance, where it is expanded.**

**R10)** We thank the reviewer for this comment, and we have made the corresponding corrections.

**C11) In the discussion, I appreciate the mention of challenges female older adult Argentinians face. That being said, mention of more older-adult specific challenges would be beneficial. For instance, lower functional independence, lack of social supports, etc. These are generally well recognized in the literature. As it stands, the discussion primarily touches upon covid 19, confounding, and gender inequality. While all important, these appear quite general and could apply to any population of study, not specifically to older adults taking antipsychotics.**

**R11)** We thank the reviewer for this thoughtful comment. We agree that incorporating additional discussion of challenges specific to older adults could enhance the contextual understanding of our findings. We have now briefly acknowledged them in the Discussion to strengthen the connection between our results and the broader geriatric context.

Regarding the second part of the comment, we agree that factors such as gender inequities and the COVID-19 pandemic affected the general population. However, these conditions may have had a particular impact on older adults treated with antipsychotics, who depend more heavily on healthcare access and support networks for monitoring and managing adverse effects. We have added a comment noting that these contextual factors could have contributed specifically to the association observed in this population in the Discussion section.

**Changes in manuscript**

**Line 350,** add: “*As in many aging populations, older adults in Argentina often experience progressive loss of functional independence and social support networks, due to biological changes and life-course transitions (e.g., retirement). Previous studies suggest that demographic shifts (e.g., declining birth rates) may have reduced the availability of informal caregiving, potentially increasing caregiver burden (31) (32). In resource-constrained settings like Argentina, these circumstances are frequently accompanied by economic vulnerability. Specifically, among women, lifelong gender inequities (e.g., lower education, wage gaps) have led to precarious retirement conditions and greater economic dependency on male partners (30). Collectively, these factors may contribute to insufficient care and limited access to non-pharmacological interventions*.”

**Line 363,** add: “*In the Buenos Aires Metropolitan Area, were most participants of this study potentially resided (9), the country implemented the strictest lockdown and social distancing measures (34). These restrictions may have had an even greater impact on older adults receiving antipsychotic treatment, who are particularly vulnerable to disruptions in care (35).”*

**C12) Line 344: ‘Finally, the strong association between antipsychotic use and mortality in this study may reflect unmeasured or residual confounding”. This statement is too general, as all studies have the potential for confounding. It would be good to either remove this sentence if the authors feel like there are not particularly poignant potential confounders readers should know about, or if there are, to move this to the limitations where it is discussed in more detail.**

**R12)** We thank the reviewer for this comment; following their advice, we remove this phrase from the manuscript.

**Change in manuscript**

**Line 367,** remove: “*Finally, the strong association between new-use of antipsychotics use and mortality in this study may reflect unmeasured or residual confounding (36). However, we included several confounding variables in our model, including education and depressive symptom scales, which are seldom available in most observational studies using claims-based datasets.”*

**Comments for the editorial board**

We have made available an adapted version of R code in a public repository alongside a mock dataset to provide detailed technical details of the analysis implementation.

**Changes included to improve readability and correct typos or minor mistakes:**

**Line 61,** add: “*cumulative”.*

**Line 62,** add: *“…the aHRs were inconsistent…”*

**Line 63,** add: “*minimizing the use of”.*

**Line 107, 108,** remove “*potentially”*; add: “*limited datasets with potentially identifiable information”*

**Line 159,** replace “until” with “*to*”.

**Line 228,** add: “cancer”, “chronic obstructive pulmonary disease”.

**Line 385,** replace “Nonetheless” with “*Nevertheless*”.
